# Non-typhoidal *Salmonella* transmission reservoirs in sub-Saharan Africa: a genomic assessment from a One Health perspective

**DOI:** 10.1186/s13756-025-01561-2

**Published:** 2025-05-13

**Authors:** Thorsten Thye, Ralf Krumkamp, John P. A. Lusingu, Linda Aurelia Ofori, Daniel T. R. Minja, Antje Flieger, Samwel Gesase, Richard Phillips, Sandra Simon, Kwasi Obiri-Danso, Charity Wiafe Akenten, Joyce Mbwana, Ellis Paintsil, Oumou Maiga Ascofare, Anna Jaeger, Maike Lamshöft, Daniel Eibach, Wibke Loag, Stefan Berg, Jürgen May, Denise Dekker

**Affiliations:** 1 Department of Infectious Disease Epidemiology, Bernhard Nocht Institute for Tropical Medicine (BNITM), Bernhard-Nochtstr. 74, 20359 Hamburg, Germany; 2 German Centre for Infection Research (DZIF), Hamburg-Lübeck-Borstel-Riems, Inhoffenstr.7, Brunswick, 338124 Germany; 3 National Institute for Medical Research (NIMR), Bombo Rd., Tanga, Tanzania; 4 Department of Theoretical and Applied Biology, Kwame Nkrumah University of Science and Technology (KNUST), KNUST Campus, Kumasi, Ghana; 5 Robert Koch Institute (RKI), National Reference Center for Salmonella and Other Bacterial Enteric Pathogens, Burgstraße 37, 38855 Wernigerode, Germany; 6 Kumasi Centre for Collaborative Research in Tropical Medicine (KCCR), Asnogya Rd., Kumasi, Ghana; 7 One Health Bacteriology Research Group, Bernhard Nocht Institute for Tropical Medicine (BNITM), Bernhard-Nochtstr. 74, 20359 Hamburg, Germany

**Keywords:** *Salmonella enterica*, Human-animal-environmental interface, Pathogen reservoirs, Drug resistance, Tropical Africa, Molecular epidemiology

## Abstract

**Background:**

In sub-Saharan Africa, invasive non-typhoidal *Salmonella* disease, characterized by bloodstream infections with high mortality rates, poses a significant public health burden. In Africa, *Salmonella enterica*, which are typically livestock- associated pathogens in industrialised countries, have genetically evolved and anthroponotic transmission has been proposed for *S*. Typhimurium ST313. In this study, we investigated the hypothesis of an exclusively anthroponotic transmission reservoir of *Salmonella enterica* ST313 and aimed to identify reservoirs for other *Salmonella* spp*.*, shedding light on their occurrence in different ecological niches.

**Methods:**

This study used a One Health approach and *Salmonella* were isolated from humans, livestock and the environment, in Tanzania and in Ghana. *Salmonella* spp. were identified by biochemical methods and antibiotic susceptibility was tested. Isolates were subjected to whole genome sequencing.

**Results:**

Out of 9,086 collected samples, 222 *Salmonella enterica* were identified comprising 58 serovars. The highest level of antimicrobial resistance was found in humans with emerging fluroquinolone resistance and multidrug resistance being highest in isolates from blood cultures (24%, n/N = 11/46). For the invasive strains, the sequence types *S*. Typhimurium ST313 and ST19 were most common and ST313 was associated with multidrug resistance, followed by *S*. Enteritidis ST11 and ST147 and *S*. Dublin ST10. An overlap of sequence types amongst human-livestock and human-environmental strains was detected for *S*. Typhimurium ST19 but not found for ST313 and the two serovars Dublin and Enteritidis.

**Conclusions:**

Our study adds further evidence of *S*. Typhimurium ST313 being restricted to a human reservoir and linked to multidrug resistance. Additionally, our study provides comprehensive insights into *Salmonella* genetic diversity and distribution among humans, animals and the environment in Ghana and in Tanzania. This sheds light on other potential reservoirs for infections, all of which show antimicrobial resistance. Further research into stool carriage is warranted, encompassing patients with invasive disease and those with and without diarrhoea, to identify transmission reservoirs in particular for invasive disease-causing strains. These findings underscore the need for integrated One Health approaches to effectively monitor and manage salmonellosis and mitigate public health risks. Continued research into the spread of *Salmonella* spp. and its evolution is crucial for targeted interventions and disease control.

**Supplementary Information:**

The online version contains supplementary material available at 10.1186/s13756-025-01561-2.

## Background

### Burden and epidemiology of non-typhoidal *Salmonella* in sub-Saharan Africa

Globally, infections with non-typhoidal *Salmonella* (NTS) are estimated to be responsible for more than 93 million enteric infections and 3.4 million cases of invasive disease each year, with the majority occurring in the global South [1–3]. In industrialised countries, infections with NTS are typically restricted to gastrointestinal disease [4]. In contrast, in sub-Saharan Africa (SSA), NTS are the most frequent cause of bacterial bloodstream infections in both adults and children, associated with high case fatality rates [5, 6]. The burden of invasive NTS (iNTS) disease in Africa is substantial in particular affecting children aged below five years [7, 8]. In this age group, the disease incidence stands at approximately 227 cases per 100,000 population, with estimated 1.9 million cases and 68,100 annual deaths [3, 7]. Important risk factors are malaria, anaemia and malnutrition [9] and infection with Human Immunodeficiency Virus (HIV) [10]. In Africa and industrialized countries, *Salmonella enterica* serovars Typhimurium and Enteritidis cause the majority of NTS infections [11–13]. In SSA, *S*. Typhimurium is the most common cause of all iNTS disease [13] being responsible for approximately two-thirds of iNTS infections [14], therefore deserving particular attention. The majority of these, belong to multilocus sequence type (MLST) 313 (ST313) with three distinct lineages (L1, L2, L3), of which both L1 and L2 are associated with multidrug resistance (MDR), hence conferring resistance to ampicillin, trimethoprim/sulfamethoxazole and chloramphenicol [14–16]. Over the past decade, the prevailing lineage among isolated invasive *S*. Typhimurium ST313 has been lineage 2 [9]. The success of these African lineages is thought to be attributed to increased antimicrobial resistance (AMR) and the ascendence of HIV [15]. Nevertheless, not all infections with this serovar are due to ST313. In other parts of Africa, even if less frequent, *S*. Typhimurium ST19 has been reported as a cause of invasive disease [1]. In addition, distinct *S*. Enteritidis lineages of ST11, also associated with MDR, are a common cause of iNTS disease in SSA [1].

### Zoonotic and anthroponotic transmission pathways

In the global north, NTS infections are typically of zoonotic origin [17, 18] often linked to food-borne outbreaks. A broad spectrum of animal food products such as poultry, beef, pork, and eggs [19, 20] and contact with farm animals [21] have been associated with infections. *Salmonella* serovar Enteritidis is strongly linked to poultry farming and egg production [22], however food-associated *Salmonella enteric*a outbreaks and zoonotic transmission in SSA are rarely investigated. So far, studies from Africa on *Salmonella enterica* isolated from livestock, animal products and environmental samples demonstrate a rather broad serovar distribution of types not commonly associated with human infections [23–26]. In a study by Kariuki and colleagues, NTS serovars isolated from humans could not be linked to strains isolated from animal sources [27]. Furthermore, a zoonotic origin for ST313 infections has not been found by other studies, suggesting anthroponotic transmission as the major transmission route of these distinct African *Salmonella enterica* lineages [28].

### Genomic adaptation

Whole genome sequencing (WGS) data of human iNTS from SSA revealed genome degradation including chromosomal deletions and pseudogene formation in the *S*. Typhimurium ST313 and in *S*. Enteritidis isolates [2, 9, 29, 30]. The authors proposed that the apparent genomic degradation is directed to specific pathways needed for survival in the inflamed intestine. Observed adaptations include for example loss of metabolites reducing fitness in the inflamed gut and downregulation of flagellin, enabling evasion of the immune system and thus facilitating invasion [2, 30]. This observation strongly suggests that the genome of the recently evolved African *Salmonella enterica* has adapted within specific hosts with some parallels observed in the *Salmonella* serovars Typhi and Paratyphi A, both solely transmitted anthropologically [31, 32]. The assumption that the recently evolved African strains are transmitted from humans to humans just like *Salmonella* of the serovars Typhi and Paratyphi is further supported by a study that was investigating human stool excretion of *Salmonella* causing invasive disease. The study showed that a proportion of children with iNTS disease were excreting NTS with high genetic similarity in their stools. The same was found in asymptomatic carriers, hence adding evidence of humans as potential reservoirs [1, 33]. Existence of *Salmonella* serovars, which are specifically adapted to a human or zoonotic reservoir, with no or limited potential to be transmitted outside of this reservoir, would explain why anthroponotic transmission is likely to be the major transmission route of the recently evolved African *Salmonella enterica*. There is an important information gap, which urgently needs to be filled to understand infection reservoirs, *Salmonella* host adaptation, as well as transmission pathways in order to devise effective management and control strategies.

In the present study we investigated potential anthroponotic transmission of the evolved African *Salmonella* Typhimurium and we aimed to identify transmission reservoirs of other NTS, shedding more light on bacterial adaptation within specific host environments. Focusing on Ghana and Tanzania and extensive sampling we examined the molecular epidemiology of *Salmonella* spp. across diverse ecological niches, significantly contributing to a deeper understanding of NTS dynamics in SSA. We employed a One Health approach in order to recognize the interface of humans, animals and the environment when tackling disease.

## Methods

### Study sites

The study was conducted in the two SSA countries: Ghana and Tanzania. Patients were recruited at the Agogo Presbyterian Hospital (APH) in the Asante Akim North District of Ghana and at the Korogwe Town Hospital (KTH) situated in the Tanga Region of Tanzania. Both hospitals serve rural areas. Livestock and environmental sampling were conducted in the respective hospital catchment areas of the Asante Akim North District and the Korogwe District of the Tanga Region.

### Study participants and sample collection

The same procedures and methods were used in Ghana and in Tanzania.

Stool samples were exclusively collected from children aged < 6 years who had experienced three loose stools within a single day during the last three days and were currently attending the Outpatient Departments of APH and KTH for care. In addition, samples were collected from children without diarrhoea of the same age group attending the vaccination clinics of the same study hospitals. Samples were collected in sterile stool collection containers and transported to the laboratory in a cool box within 1–3 h.

For blood culture collection, 1–3 mL venous blood was collected from children aged < 6 years with at least one of the following inclusion criteria; suspected sepsis, fever (≥ 37.5), or a history of fever within the past 48 h and admitted to the respective study hospital. The patient’s blood was drawn into Becton Dickinson (BD) BACTEC® Peds Plus Medium.

### Livestock sampling

Individual faecal samples from commercial farms and smallholder farms were collected, including poultry, cows, pigs, sheep and goats. Priority was given to poultry, followed by cows and pigs then sheep and goats. In addition, pooled faecal samples from chicken in commercial- and smallholder farms consisting of ten individual samples were collected from pens. A sterile plastic container was used for collection and samples were transported to the laboratory within 1–4 h using a cool box.

### Environmental sampling

Dust samples were collected from fences and doors of farms as well as from animal feeding/water troughs using sterile, saline moistened surgical head caps. Each sample was placed in a labelled sterile plastic container and transported in a cool box to the laboratory within 1–4 h of collection.

Soil was taken close to farms and households. For sampling, a soil core sampler was used [34]. The soil was then collected using a plastic spoon. Pieces of equipment were disinfected in between sample taking. For each sample, 10 g was placed into a sterile plastic zip lock bag and transported to the lab in a cool box within 1–4 h of collection.

### Bacterial culture and identification

Blood cultures were processed using a BACTEC® 9050 blood culture system (Becton Dickinson, USA) according to manufacturer’s instructions. For positive blood cultures, aspirated blood culture fluid was Gram stained for preliminary identification and inoculated on Columbia blood-, chocolate-, and MacConkey agar (all Oxoid, Basingstoke, UK). Plates were incubated at 35–37 °C for 18–24 h in normal atmosphere.

Human and animal stool samples as well as environmental samples were enriched using Selenite broth (Oxoid) and after 18–24 h incubation in normal atmosphere plated on Xylose Lysine Deoxycholate agar (XLD agar from Oxoid). Plates were incubated for 18–24 h in normal atmosphere.

Bacterial strains were identified by colony morphology, Gram stain and standard biochemical methods and stored in microbanks at −80 °C until transportation to Germany on dry ice for further analyses.

### *Salmonella* species typing methods

*Salmonella* subspecies were determined by biochemical reactions e.g., visualizing carbon source metabolism. Serotyping was performed by slide agglutination according to the White-Kauffmann-Le Minor scheme with antisera directed against the somatic (O-), flagellar (H-) and capsular (vi) antigens (SIFIN, Berlin, Germany). For the determination of flagellar antigens swarming motility was induced using inhouse-prepared Sven Gard soft agar.

### Antibiotic susceptibility testing

Susceptibility to antibiotics was tested by the broth microdilution method and interpreted following the European Committee on Antimicrobial Susceptibility Testing (EUCAST) guidelines v.12 (http://www.eucast.org). Quality control of susceptibility testing was performed according to EUCAST (QC table v.5). *Salmonella enterica* were tested against a panel of antibiotics: ampicillin, cefotaxime, cefoxitin, ceftazidime, chloramphenicol, ciprofloxacin, colistin, meropenem, nalidixic-acid and trimethoprim/sulfamethoxazole. Isolates resistant to ampicillin, chloramphenicol, and trimethoprim/sulfamethoxazole were considered MDR.

### Statistical analyses

**Fig. 1:**
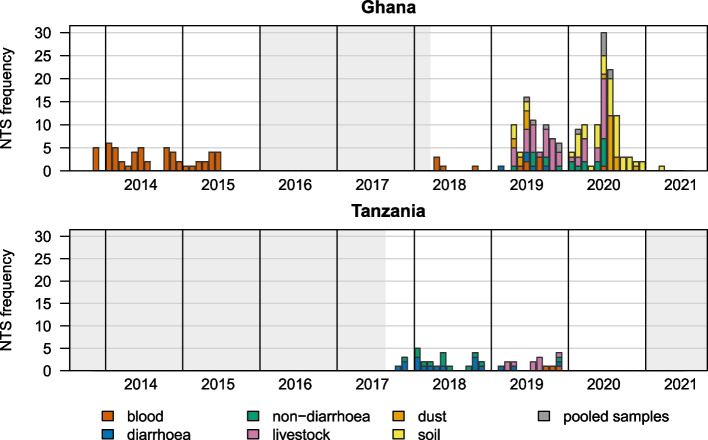
Frequency of non-typhoidal *Salmonella enterica* over time by sampling group for Ghana and Tanzania. Periods in which no sampling took place are marked as grey shaded areas

**Fig. 2:**
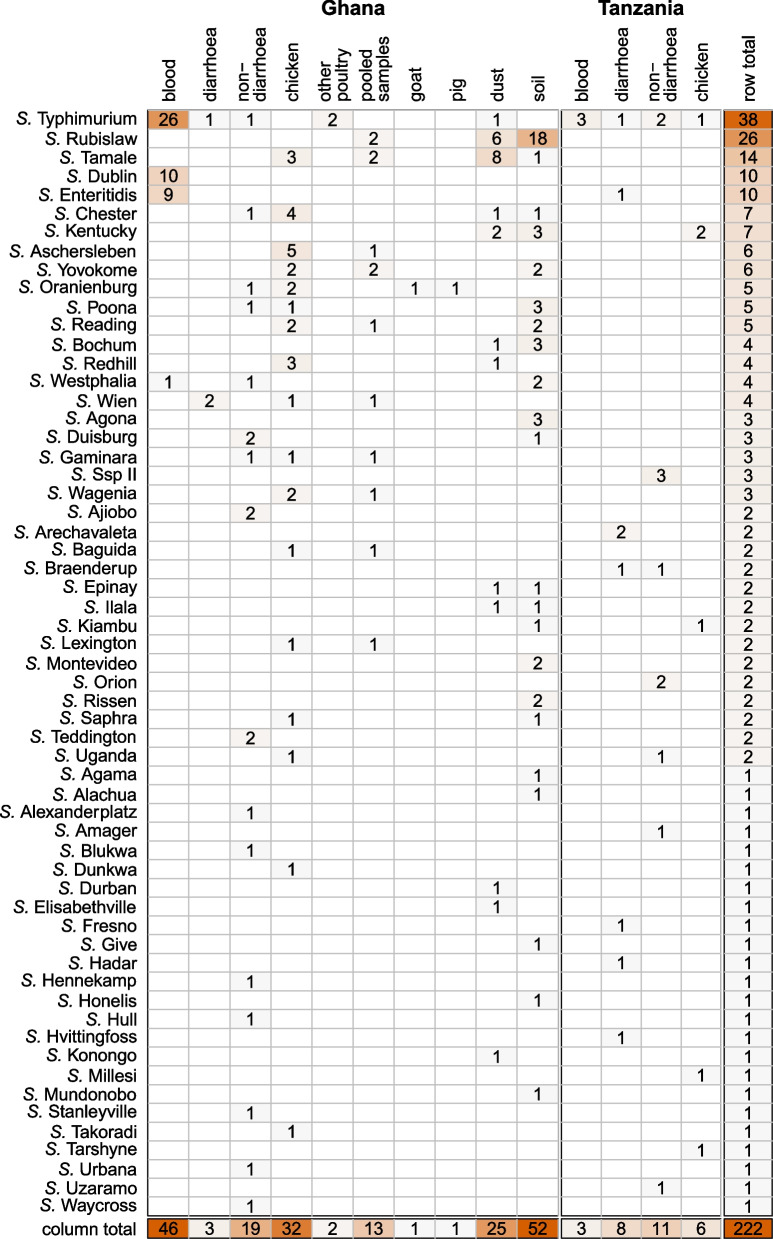
Frequency of *Salmonella* serovars by sampling group for Ghana and Tanzania

Categorical variables were described using frequencies and their proportion and continuous variables using the median and the interquartile range (IQR). Missing values (e.g., bacterial isolates lost during culture) were excluded from the analysis; hence, in some calculations, the denominator differs. Statistical analyses were conducted, and Figs. 1 and 2 were generated using base *R* (version 4.1.3).

### Whole-genome sequencing

*Salmonella* genomic DNA was extracted using the GenElute™ Bacterial Genomic DNA Kit from Sigma-Aldrich according to the manufacturers protocol. Library preparation (Illumina Nextera XT Library Preparation Kit), short-read sequencing (2 × 150 bp; Illumina NextSeq 2000) and subsequent quality control of sequence data was performed at the Sequencing Core Facility within the Genome Competence Centre at RKI.

### Bioinformatic analyses

#### Data processing and quality control

Quality of raw sequencing data was checked with the AQUAMIS software 1.2.0, which implements FASTP v0.19.5 for trimming [35], SHOVILL v1.1.0 (https://github.com/tseemann/shovill) for assembly, reference search using MASH v2.2.2, and assembly quality analysis with QUAST v5.0.2 (https://github.com/ablab/quast). In addition, contamination checks of the assembled bacterial genomes were done with CONFINDR v0.7.1 (https://github.com/OLC-Bioinformatics/ConFindr) and CHECKM2 (https://github.com/chklovski/CheckM2).

#### Pan-genome analysis

Assembled contigs were annotated using BAKTA v1.14.6 (https://github.com/oschwengers/bakta). The core genome was determined using PANAROO v3.13.0 (https://github.com/gtonkinhill/panaroo), taking GFF files with default settings. A core genome alignment was done with MAFFT (v7.467) (https://mafft.cbrc.jp/alignment/software/).

#### Phylogeny construction

All 222 assembled *Salmonella enterica* genomes were compared on a whole genome scale using core genome MLST (cgMLST) screens with CHEWIESNAKE (https://gitlab.com/bfr_bioinformatics/chewieSnake) using the allele calling algorithm CHEWBBACA (https://github.com/B-UMMI/chewBBACA). To infer alleles, the *S. enterica* cgMLST scheme of the Enterobase database (https://enterobase.warwick.ac.uk) was applied. A maximum likelihood phylogenetic tree was computed with the IQTREE (http://www.iqtree.org) software.

For a more detailed comparison of the 38 (*n* = 11, ST313; *n* = 27, ST19) *S.* Typhimurium samples, a SNP cluster analyses was done with *S.* Typhimurium isolates only. The SNIPPYSNAKE pipeline was therefore used to assess SNP variant calling of trimmed reads. The optimal reference for SNP calling was calculated with the ‘reffinder’ module of the pipeline. Phylogenies were constructed with IQTREE.

Because of their invasive potential *S.* Typhimurium ST19 and ST313 found in this study were brought into global context by comparison of strains with ST19/ST313 genomes extracted from the NCBI reference databases. Therefore all *S.* Typhimurium assemblies from the NCBI nucleotide database were downloaded, typed by MLST and selected according to their ST19 or ST313 profiles. After quality controls of 22,000 ST19 assemblies, 19,579 remained excluding isolates with N50 < 50 kb, contamination values > 5% and contigs numbers > 500. The number of 19,579 was reduced by similarity mapping with a MASH value of 0.0015 by applying the Assembly-Dereplicator tool (https://github.com/rrwick/Assembly-Dereplicator) resulting in 165 ST19 assemblies plus 27 ST19 isolates of the present study.

For the ST313, only 815 genome datasets were identified in the NCBI reference database resulting in 41 data sets after quality checks and dereplication using a MASH threshold of 0.0005. The assemblies were annotated with BAKTA v1.14.6, and a core gene alignment including the genome data of all *S.* Typhimurium strains of the present study was constructed for the ST19 and ST313 genotypes with the PANAROO software. For both sequence types SNP-SITES was used to create a core SNP alignment which was taken to construct a maximum likelihood phylogeny with IQTREE. Visualization of trees was performed with the R library ggTREE.

#### In silico antimicrobial resistance, plasmid replicon, and virulence factor detection

AMR determinants were identified within the 222 *Salmonella* genome assemblies, using STARAMR v0.0.1 (https://github.com/phac-nml/staramr). The software makes use of the latest version RESFINDER database. The presence of virulence factors in the genome assemblies was analysed with ABRICATE v1.0.1 (https://github.com/tseemann/abricate) implementing the VFDB database (https://github.com/tseemann/abricate). Screening for the presence of plasmids was also performed applying the MOB-SUITE software package (https://github.com/phac-nml/mob-suite) using the mob-cluster algorithm.

#### Screening for genome variations characteristic for ST313 and ST19 sequence types

To assess genome variations between the ST19 and ST313 and to compare ST313 strains identified in Ghana and Tanzania to those analysed in a study from Malawi [14], assembled genomes of the present study were screened for the presence of the specific genes *ratB*, *katE*, *ttdA*, *macB*, *melR*, *flhA*, *pipD*, *bcsG*, *sseI*, and *lpxO* known to be occurring in different combinations in lineages L1-3 of ST313 and also the ST19 sequence type [9]. The occurrence of bacteriophages which were found to correlate with the emergence of ST313 was investigated by the PHASTEST (PHAge Search Tool with Enhanced Sequence Translation; https://phastest.ca) screening tool and also BLAST searches in *Salmonella* strains from Ghana and Tanzania.

#### Co-occurrence of genes

Gene co-occurrence analysis in bacteria allows the identification of patterns of gene associations and deducing potential functional relationships. The co-occurrences of specific genes of all strains were computed using COINFINDER package). (https://github.com/fwhelan/coinfinder). The tool detects genes, which associate and dissociate with other genes more often than expected based on a presence/absence matrix of the pan-genome generated with the PANAROO software. A stringent significance cut-off of e-12 for the binomial exact test followed by Bonferroni’s correction was chosen.

## Results

This study was conducted in two sub-Saharan African countries; Ghana and Tanzania. Data was collected using a One Health approach from humans, livestock and the environment. In total, we analysed 9,086 samples. Table 1 provides an overview of the sampling dates, characteristics of individual sampling categories and the NTS positivity rate for the different sampling groups. Blood samples in Ghana were collected over two periods, one ranging from November 2013 to December 2015 and another from April 2018 to July 2020. Median age of febrile children was 2 years (IQR: 1–4). In total 2,231 blood culture samples were collected, of which 67 (3%) tested positive for NTS. In Tanzania, blood samples from febrile children were collected between March 2019 and December 2020, and 637 observations were analysed. Children had a median age of 2 years (IQR: 1–3) and, compared to Ghana, NTS bacteraemia was rather low (n/N = 3/637; 0.5%).

**Table 1:** Sample and patient characteristics included in the study from Ghana and Tanzania

	Blood	Diarrhoea	Non-Diarrhoea	Livestock faecal samples	Pooled faecal samples	Dust	Soil
Ghana							
Number of samples	2,231	376	585	1,570	409	281	801
Sampling date [range]	Nov 2013 – Jul 2020	Oct 2018 – Jul 2020	Nov 2018 – Jun 2020	Apr 2019 – Jun 2020	Apr 2019– Nov 2020	Apr 2019– Nov 2020	Apr 2019– Jun 2021
Age (years) of participants [median (IQR)]	2 (1–4)	1 (0–2)	2 (1–4)	NA	NA	NA	NA
Female sex [n (%)]	969 (43)	166 (44)	298 (51)	NA	NA	NA	NA
NTS detection [n (%)]	67 (3)	5 (1)	21 (4)	56 (4)	13 (3)	25 (9)	52 (6)
Isolate available [n (%)]	46 (69)	3 (60)	19 (90)	36 (64)	13 (100)	25 (100)	52 (100)
Tanzania							
Number of observations	637	575	574	1,047	0	0	0
Sampling date [range]	Mar 2019 – Dec 2020	Sep 2017 – Jul 2020	Sep 2017 – Jul 2020	Mar 2019 – Jul 2020	NA	NA	NA
Age (years) of participants [median (IQR)]	2 (1–3)	1 (0–1)	1 (0–1)	NA	NA	NA	NA
Female sex [n (%)]	279 (44)	268 (47)	272 (47)	NA	NA	NA	NA
NTS detection [n (%)]	3 (0)	17 (3)	13 (2)	9 (1)	NA	NA	NA
Isolate available [n (%)]	3 (100)	8 (47)	11 (85)	6 (67)	NA	NA	NA

Stool samples from children with diarrhoea were collected in Ghana between October 2018 and July 2020, and in Tanzania between September 2017 and July 2020. Five (1%) of the 376 samples collected in Ghana and 17 (3%) of the 575 samples collected in Tanzania tested positive for NTS. This can be compared with stool samples from children without gastrointestinal symptoms, which were collected during similar periods: 21 (4%) of the 585 samples collected in Ghana and 13 (2%) of the 574 samples collected in Tanzania were NTS positive, respectively.

In both countries, livestock faecal samples from poultry, pigs, sheep, goats and cows were collected between April 2019 and June 2020. In Ghana 1,570 and in Tanzania 1,047 samples were collected, of which 56 (4%) and 9 (1%) were NTS positive, respectively. In Ghana, also 409 pooled faecal samples from chicken were collected at commercial farms of which 13 (3%) were NTS positive.

In Ghana, also environmental samples were collected including 281 dust and 801 soil samples. Of these, 25 (9%) and 52 (6%), respectively were NTS positive.

Figure 1 summarizes the frequency of NTS over time for the different sampling groups. The figure highlights that most NTS were detected in Ghana, which also include samples collected from the environment (dust and soil) as well as samples collected during an earlier study in Ghana on febrile children.

### Serovar distribution amongst the different sampling groups

Of the 281 detected NTS isolates, 222 (79%) were available for further analysis. Figure 2 shows frequencies of the different NTS serovars by sampling group and country, highlighting serovar diversity among the studied categories for sampling. In the following paragraphs, the serovar distribution among the groups is described.

### Blood samples

From 70 *Salmonella* isolates detected in blood samples of febrile children, 49 were available for further analysis. Four different serovars were detected, namely *S.* Typhimurium (*n* = 29; 59%), *S.* Dublin (*n* = 10; 20%), *S.* Enteritidis (*n* = 9; 18%) and *S.* Westphalia (*n* = 1; 2%). Forty-six iNTS isolates were from Ghana, 35 of them from samples collected during the 2013–2015 study period (*n* = 35, 71%). All three isolates from Tanzania were *S*. Typhimurium.

### Stool samples from children with diarrhoea

In total, 11 isolates from stool samples from children with diarrhoea were available, of which 8 (73%) were from Tanzania. The serovar distribution was diverse and 8 different serovars were detected. Only *S.* Arechavaleta (all from Tanzania), *S.* Typhimurium (Ghana and Tanzania) and *S.* Wien (both from Ghana) were detected twice, while the other three serovars were represented by single samples.

### Stool samples from children without diarrhoea

Thirty isolates from samples of children without diarrhoea were available for analysis. Similar to the samples from children with diarrhoea, the serovar spectrum was diverse with a total of 22 different serovars. *S*. Typhimurium was the only serovar detected thrice, once in Ghana and twice in Tanzania.

### Livestock samples

In Ghana 1,570 livestock samples were collected, 1,206 (81%) from chicken, 28 (2%) from quails, 27 (2%) from turkey, 26 (2%) from ducks, 110 (7%) from goats, 83 (5%) from pigs, 65 (4%) from cows and 25 (2%) from sheep. In the following, quails, turkeys and ducks are summarised into the category “other poultry”. In total 34 *Salmonella* isolates were detected, the majority (*n* = 32; 94%) in chicken, which was also the largest livestock group. The serovar distribution in chicken was diverse and 17 different *Salmonella* serovars were detected. Serovar types with at least three observations were *S.* Aschersleben (*n* = 5; 16%), *S.* Chester (*n* = 4;13%), *S.* Redhill (*n* = 3; 9%), and *S.* Tamale (*n* = 3; 9%). Furthermore, two *S*. Typhimurium isolates were detected in other poultry, one in a turkey (ST19) and one in a duck (ST19).

In addition, pooled faecal chicken samples (*N* = 409) were available from Ghana. Thirteen (3%) *Salmonella* were detected and they belonged to 10 different serovars. Serovars detected twice were *S.* Rubislaw, *S.* Tamale and *S.* Yovokome. Other serovars were detected once each. Furthermore, in one sample each from goats and pigs, *S.* Oranienburg was detected*.*

In Tanzania 1,047 livestock samples were collected, of which 777 (40%) were from chicken, 206 (11%) from cows, 23 (1%) from goat, 39 (2%) from pigs and 2 (0%) from sheep. Six *Salmonella* isolates were found in chicken. Five different serovars were detected and *S.* Kentucky (*n* = 2, 40%) was the only serovar detected twice.

### Environmental samples

In Ghana environmental samples were collected from dust (*N* = 281) and soil (*N* = 801). In dust samples 25 (9%) *Salmonella enterica* were detected, belonging to 12 different serovars. Serovars detected in more than 2 samples were *S.* Tamale (*n* = 8; 32%) and *S.* Rubislaw (*n* = 6; 24%).

In soil samples, 52 (6%) *Salmonella enterica* were detected comprising 22 different serovars. Most frequent serovars were *S.* Rubislaw (18; 35%) followed by *S.* Agona (*n* = 3; 6%), *S.* Bochum (*n* = 3; 6%), *S.* Kentucky (*n* = 3; 6%), and *S.* Poona (*n* = 3; 6%). The serovar distribution varied widely amongst the different sampling groups. Serovars which were found in humans (blood, diarrhoea, or non-diarrhoea samples), livestock as well as in environmental samples (dust or soil) were *S.* Chester*, S.* Poona and *S.* Typhimurium. Serovars found in human and livestock samples were *S*. Gaminara, *S.* Oranienburg, *S.* Uganda and *S*. Wien, and serovars found in human and environmental samples were *S.* Duisburg and *S.* Westphalia.

### Antimicrobial resistance of *Salmonella enterica*

#### Blood cultures in Ghana and Tanzania

In Ghana the highest frequency of resistance seen was for trimethoprim/sulfamethoxazole (n/N = 12/49; 24%) followed by ampicillin (n/N = 11/49; 22%) and chloramphenicol (n/N = 11/49; 22%). Among isolates from Tanzania, no AMR was observed.

MDR was highest in isolates from Ghanaian blood cultures in the 2013–2016 study period (n/N = 11/35; 31%). Ten of the MDR isolates were the *S*. Typhimurium ST313 and one *S*. Enteritidis ST11. In the study period 2018–2020 none of the *Salmonella* strains from Ghana were MDR. All *S*. Dublin ST10 were resistant to colistin.

Low-level fluoroquinolone resistance was detected in 7% (n/N = 3/46) of isolates from Ghana, two in the study period 2013–2016 and one in the latter. This resistance was restricted to the serovar Enteritidis only.

#### Children with and without diarrhoea in Ghana and Tanzania

In Ghana, AMR was only observed in isolates from children with diarrhoea and included resistance to ampicillin, chloramphenicol and trimethoprim/sulfamethoxazole with 33% being resistant to each of the antibiotics tested (n/N = 1/3). One of the isolates was MDR involving the serovar Typhimurium ST313. In Tanzania, resistance in this sample category was restricted to one isolate being resistant to fluoroquinolones (n/N = 1/8; 12%). In children from Tanzania without diarrhoea, resistance was to ampicillin only (n/N = 1/11; 9%).

#### Livestock in Ghana and Tanzania

In *Salmonella enterica* from livestock in Ghana, resistance was only to chloramphenicol in one of the pooled faecal samples (n/N = 1/13; 8%). In Tanzania, AMR was only found in isolates from chicken of the serovar Kentucky, of which one sample was MDR and two were fluoroquinolone resistant (n/N = 2/6; 33%).

Other resistances observed included 33% to ampicillin (n/N = 2/6) and to trimethoprim/sulfamethoxazole (n/N = 2/6), and 17% (n/N = 1/6) were resistant to chloramphenicol.

#### Dust and soil samples in Ghana

In environmental samples, antibiotic resistance was highest for fluoroquinolones in dust samples (n/N = 5/25; 22%) and in samples from soil (n/N = 6/52; 12%) including the serovars Epinay (*n* = 1), Chester (*n* = 2) Kentucky (*n* = 5), Montevideo (*n* = 1), Give (*n* = 1), and Ilala (*n* = 1). MDR was not detected in any of the isolates but resistance to ampicillin in isolates from dust (n/N = 3/25; 12%) and soil (n/N = 5/52; 8%) and to trimethoprim/sulfamethoxazole in *Salmonella enterica* from dust (n/N = 4/25; 16%) and soil (n/N = 3/52; 6%).

### Phylogenetic analysis of the *Salmonella enterica* collection from all sample sources

**Fig. 3:**
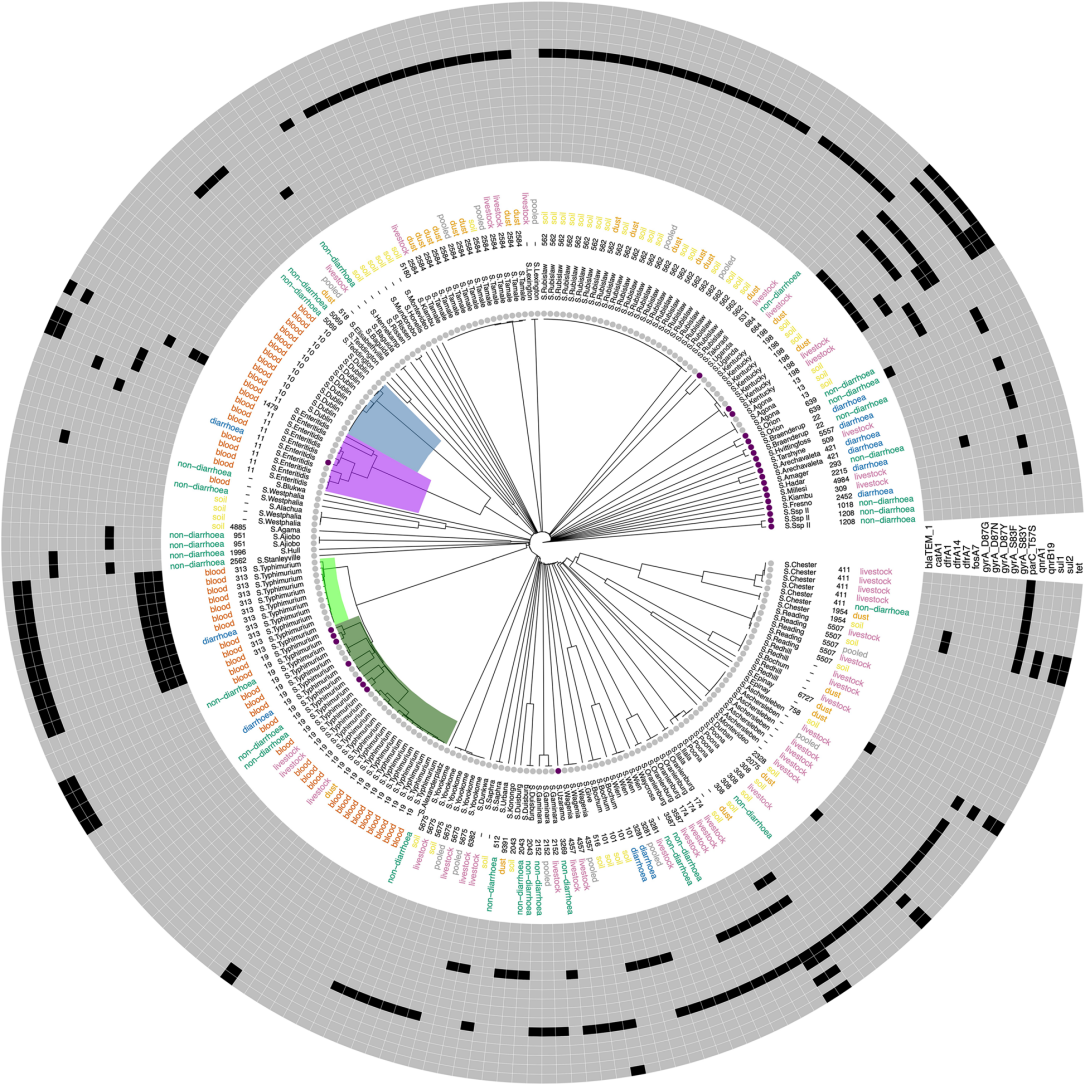
Core genome MLST-based phylogenetic tree of 222 non-typhoidal *Salmonella enterica* genomes found in Ghana and Tanzania. Descriptions from the outside to the inside: 1) Heatmap of AMR genes identified with the Resfinder software, excluding aminoglycoside resistance genes. 2) Origin of samples: human (blood, stool from children with diarrhoea, stool from children without diarrhoea); environmental samples nearby animal farms (soil, dust); livestock samples, mainly poultry (faeces); pooled faecal chicken samples (pooled). 3) MLST sequence types. 4) *Salmonella* serovars. 5) Country of origin (grey = Ghana, purple = Tanzania. 6) Clades of invasive strains coloured by sequence types: light green (ST313; *S*. Typhimurium), dark green (ST19; *S*. Typhimurium), purple (ST11, ST1479; *S*. Enteritidis), blue (ST10; *S*. Dublin)

**Fig. 4:**
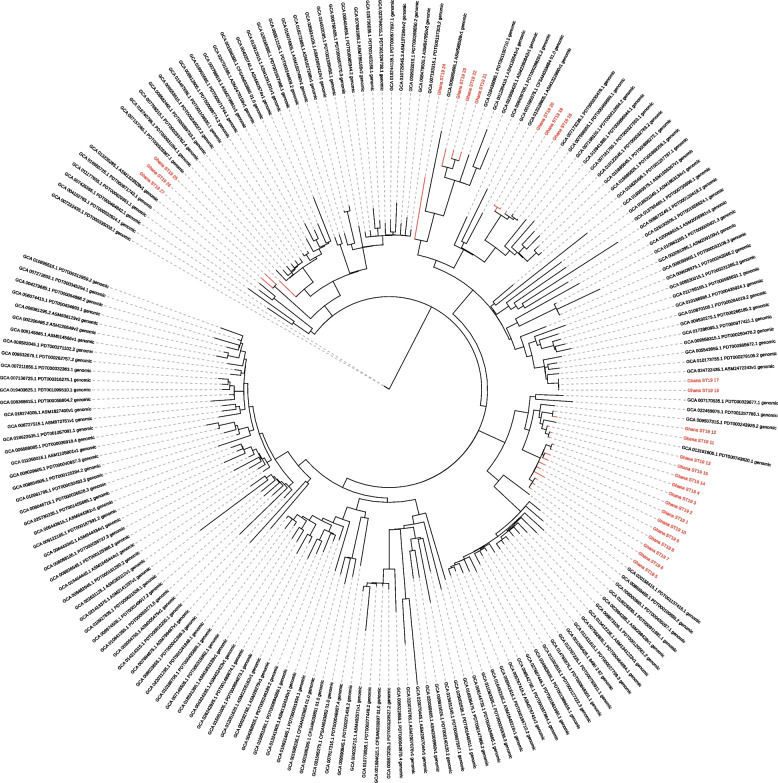
Global phylogenetic tree of *S*. Typhimurium ST19. Phylogenetic tree of *S*. Typhimurium ST19, with 27 isolates of the current study (red) combined with 165 publicly available ST19 isolates (black)

**Fig. 5:**
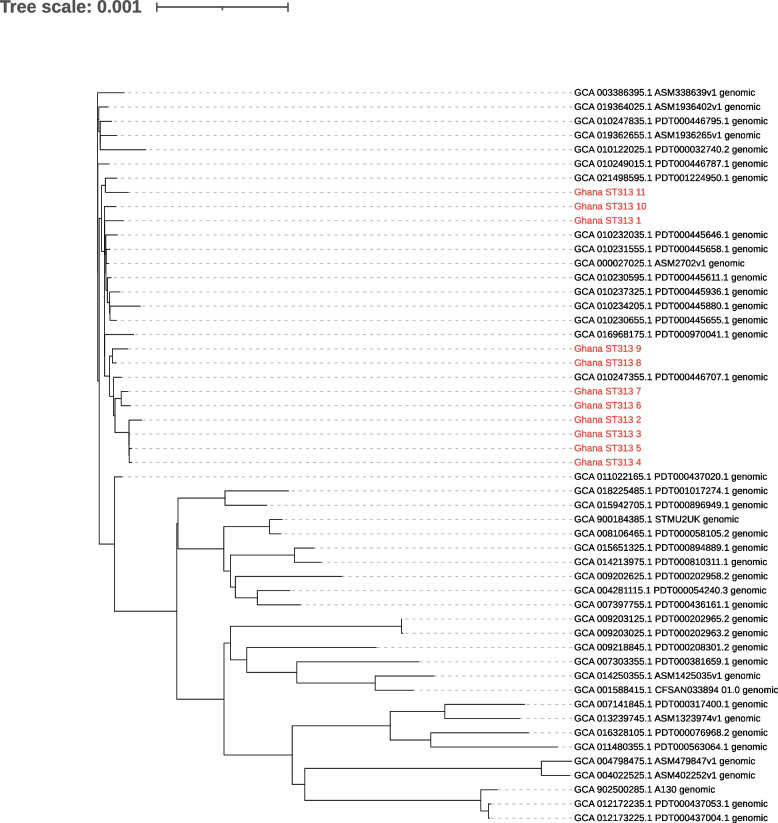
Global phylogenetic tree of *S*. Typhimurium ST 313. Phylogenetic tree of 11 *S*. Typhimurium ST313 isolates of the current study (red) combined with 41 publicly available ST313 isolates (black)

A phylogenetic tree based on cgMLST alleles was constructed (Fig. 3) to assess the relationship within the *Salmonella enterica* population isolated in this study. Three major clades with isolates of human origin were formed by the serovars *S*. Typhimurium, *S*. Enteritidis and *S*. Dublin. Of these, the majority originated from samples of invasive infections in humans but also from stool of children with and without diarrhoea. Strains of the clades *S*. Dublin (ST10) and *S*. Enteritidis (ST11, ST1479) were only found in human samples. The serovar *S*. Typhimurium was divided into subclades including the sequence types ST313, which was restricted to human stool and blood specimens, and ST19, which was found in human blood, and in children with and without diarrhoea. ST19 was also found outside the human reservoir e.g., in poultry and in samples from dust (Fig. 3). A detailed SNP-based phylogenetic classification of *S.* Typhimurium strains revealed that the ST313 isolates formed a homogeneous subclade with differences of no more than 169 SNPs between any two strains, whereas strains of the ST19 clade showed much higher diversity with SNP differences up to 561. To bring both sequence types in global context a combined phylogenetic analysis of ST19 and ST313 obtained from the NCBI assembly database (Figs. 4 and 5) was performed. Ghanaian and Tanzanian isolates from both sequence types integrated into global phylogenetic trees, showed no study-specific cluster (Figs. 4 and 5). ST313 strains were found to be phylogenetically more homogeneous with isolates of the present study found to be part of the ST313 lineage L2. It was also found that half of the *S*. Typhimurium (n/N = 10/20; 50%) were ST313 in the study period 2013–2016. ST313 was not isolated from blood cultures in the later study period but was found in one patient with diarrhoea.

Further clades with strains from human- but also from animal/environmental samples were those with the serovars *S*. Chester (ST411), *S*. Oranienburg (ST3587), *S*. Poona (ST308), *S*. Uganda (ST684) and *S*. Wien (ST3281).

Clades without strains of human origin consisting of more than five samples were mainly the environmental serovar Rubislaw (ST562), in addition to the serovars *S*. Tamale (ST2584), *S*. Yovokome (ST5675), and *S*. Aschersleben (ST unknown).

When comparing the strains between Ghana and Tanzania, four clades including the serovars *S*. Uganda (ST 684), *S*. Kentucky (ST198), *S*. Enteritidis (ST11, ST1479) and *S*. Typhimurium (ST19) were found in both countries (Fig. 3).

### Virulence factors

*Salmonella* virulence factors (VFs), including toxins, adhesins, and secretion systems play a major role in infection and disease severity. Thereby, different *Salmonella* serovars exhibited varying degrees of invasiveness, meaning their ability to invade and cause systemic infections in the host. The number of VFs differed considerably with regard to different serovars. In total, 173 VFs were found by alignment of assemblies to the VFDB database with numbers ranging from 91 to 154 per isolate with a median of 136. Clinically common *Salmonella enterica* are for example known to carry more VFs. In this study, the serovars *S.* Typhimurium, *S*. Enteritidis and *S*. Dublin having caused invasive disease carried on average more virulence factors (*N* = 146) than other *Salmonella* serovars (*N* = 134) reported in this study.

The virulence factors present in *S.* Typhimurium ST19 and ST313 were almost equally distributed (*N* = 153 vs *N* = 151) (Supplementary Table 1).

Genes of the VFDB database are categorized according to their known or putative function in 11 groups (adherence, antimicrobial, biofilm, effector, exotoxin, immune, invasion, motility, nutritional/metabolic, regulation, stress). Of the 173 VFs identified, 115 were found in at least 90% of all sequenced isolates. The majority of the VFs belonged to the ‘effector’ category with *N* = 112 (64,7%) comprising genes of the type III secretion systems. The second largest group of virulence factors identified harboured genes important for adherence to cells (*N* = 32; 18.5%). (Supplementary Table 2).

### Plasmids

Screening of assemblies revealed that not all strains carried plasmids. An analysis with the MOB‐Typer software showed that 115 of 222 isolates had no plasmids. Of those plasmid-free isolates, the largest group belonged to serovar Rubislaw. The clinically common *Salmonella* strains carried large plasmids similar to the known *Salmonella* virulence plasmid pSLT (Jones GW, 1982) of *S.* Typhimurium with several virulence factors. *S.* Enteritidis and *S.* Dublin also carried large plasmids with sizes of > 50 kb and > 60 kb, respectively.

*S.* Typhimurium ST313 also carried additional smaller plasmids known to be part of the lineage L2 (Supplementary Table 3). When analysing the gene content of plasmid sequences ST19 and ST313 strains in more detail, AMR genes were identified on the largest ST313 plasmid with high homology to the known *Salmonella* virulence plasmid pSLT (Jones GW, 1982). This cassette of resistance genes was not seen on the major plasmid of the ST19 serovar. Although the gene content of the major plasmids found in invasive strains was different in each serovar, several virulence genes of the spvA-R cluster were common in all invasive *Salmonella*. All major virulence plasmids of the invasive strains had conjugative abilities except those found in *S.* Enteritidis*,* which were non-mobilizable. When comparing incompatibility complexes of ST313 plasmid with those of ST19 strains the ST313 carried IncFIB, IncFII, IncQ1 whereas the majority of ST19 only harboured IncFIB and IncFII.

### Prophages

The analysis revealed a broad spectrum of prophages in the assembled genomes of the analysed *Salmonella*. PHASTEST (https://phastest.ca) analysis and BLAST (https://blast.ncbi.nlm.nih.gov/Blast.cgi) screens yielded 18 intact prophages in the *Salmonella* genomes of this study (Supplementary Table 4). Ninety-six strains appeared to not carry any intact prophages as assessed with the PHASTEST and BLAST software. The distribution of prophages was heterogeneous amongst the *Salmonella* serovars with the exception of *S.* Dublin, which carried solely the ST160 prophage, the *S*. Rubislaw serovar with Gifsy-1 integrated, and *S.* Typhimurium ST313 with the prophages BTP1, BTP5 and ST64B. Of the *S.* Typhimurium ST19 a large proportion of the strains carried the Gifsy-1 phage (59%).

### Gene co-occurrence analysis

**Fig. 6:**
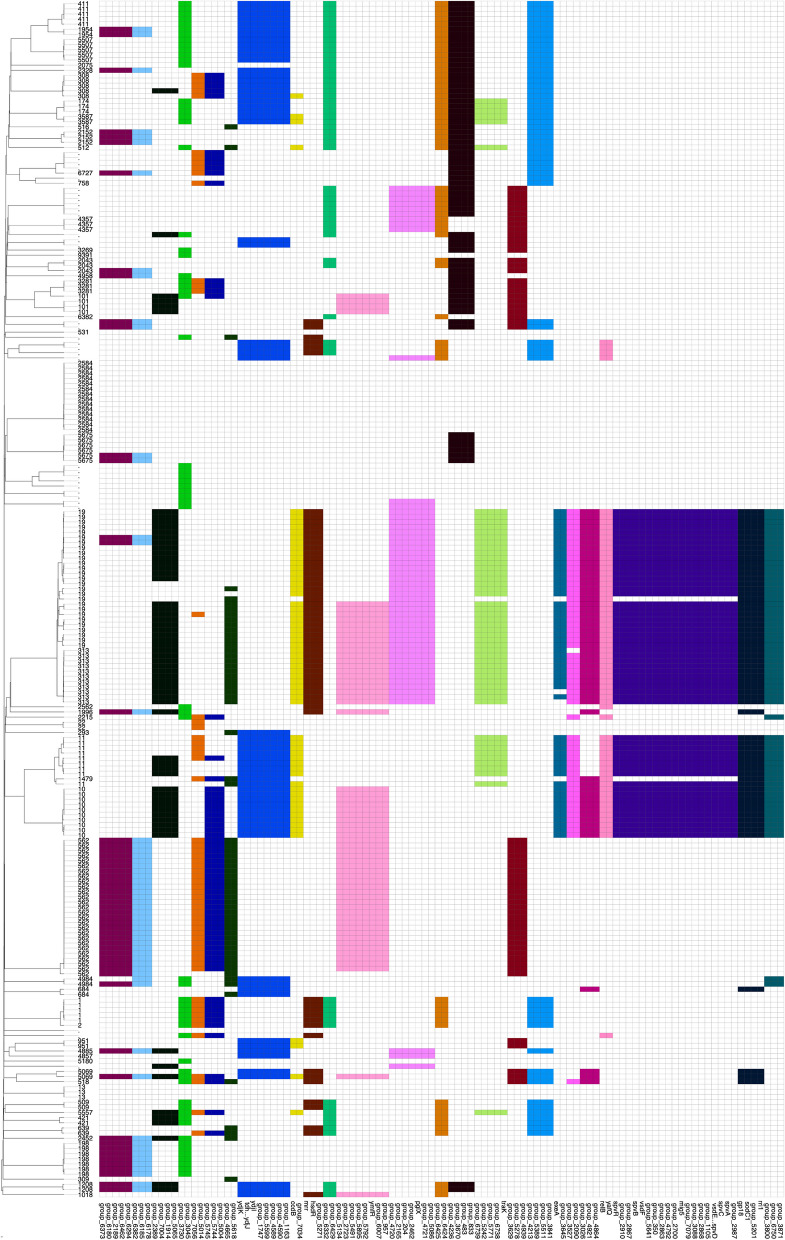
Gene co-occurrence analysis of 222 *Salmonella enterica* strains. Co-occurrence analysis was performed based on the presences/absence matrix of a pan-genome screen with all 222 *Salmonella enterica*. A significance threshold of e-12 for the binomial exact test was chosen

Coinfinder was run to test for significant co-occurrences of genes between sequence types applying a stringent significance threshold of e-12. A total of 25 co-occurring gene clusters with different gene content came up. The size of these gene clusters varied considerably from 2 to 19 genes. The largest gene cluster was shared between mainly the invasive serovars *S.* Dublin, *S.* Enteritidis and *S.* Typhimurium and contained multiple genes associated with increased virulence and are known to be part of the *Salmonella* virulence plasmid pSLT. Among these genes are the known *Salmonella* virulence factor genes *spvA*, *spvB* and *spvC*. Further heterogeneous cluster of gene–gene associations were mainly between hypothetical genes (Fig. 6).

### Genotypic antimicrobial resistance

To obtain epidemiological insights into AMR in *Salmonella enterica* from different niches such as soil, dust, livestock and humans, we investigated the dynamics of AMR. The draft genome sequences of the bacteria under investigation were examined for genes and mutations that confer reduced susceptibility to antimicrobials. In total, 114 (51,4%) of the isolates (*n* = 222) did not carry any known resistance genes or mutations as assessed with the RESFINDER software. Of the invasive serovars only 5 of the 27 ST19 (*S.* Typhimurium*)* isolates were identified to carry resistance-conferring genes, including *aph(3'')-Ib* (streptomycin), *aph(6)-Id* (kanamycin), *sul2* (sulfisoxazole) and *tet(A)* (tetracycline). The resistance genes were structured as a cassette on the plasmids of size > 90 kb. All *S.* Typhimurium ST313 were found to carry the same eight resistance genes *aph(3'')-Ib* and *aadA1* (streptomycin), *aph(6)-Id* (kanamycin), *blaTEM-1B* (ampicillin), *catA1* (chloramphenicol), *dfrA1* (trimethoprim) and *sul1* and *sul2* (sulfisoxazole) (Supplementary Table 5), all conferring MDR. *S.* Enteritidis were found carrying a heterogeneous combination of resistance genes including different point mutations in the gyrase gene *gyrA* rendering the strains less susceptible to fluroquinolones. *S*. Dublin isolates carried no genes with a known resistance phenotype. The serovar with the most resistance mutations and genes *S.* Kentucky were found in samples taken from dust, soil and from livestock. Most genes and mutations conferring resistance were found in all *S.* Kentucky samples including *aac(3)-Id* (gentamicin), *aph(3'')-Ib* and *aadA7* (streptomycin), *aph(6)-Id* (kanamycin), *blaTEM-1B* (ampicillin), *catA1* (chloramphenicol), *gyrA* (S83 F) (quinolones), *sul1* (sulfisoxazole) and *tet(A)* (tetracycline). Additional resistance mutations and genes such as ciprofloxacin resistance mutations *gyrA* mutations (D87G/D87Y) and *parC* (S80I) conferring fluoroquinolone resistance, the trimethoprim resistance gene *dfrA14* and the sulfisoxazole gene (*sul2*) were only found in some strains. (Supplementary Table 5).

### Phenotypic and genotypic concordance

Concordance rates of genotypic and phenotypic results were determined by comparing four classes of antibiotic drugs including beta-lactam antibiotics, phenicols, fluoroquinolones, and folate inhibitors (Table 2) The concordance was 100% for the antibiotics ampicillin, ciprofloxacin and trimethoprim and lowest for trimethoprim/sulfamethoxazole with 97.8% (Table 2).

**Table 2:** Phenotypic and genotypic antibiotic resistance amongst 222 *Salmonella enterica*

Antimicrobial class	Phenotypic % (n)^a^	Genotypic % (n)^a^	Concordance (%)
Ampicillin	9.9 (22)	9.9 (22)	100
Chloramphenicol	6.3 (14)	5.4 (12)	99.1
Nalidixic acid	6.8 (15)	5.0 (11)	98.2
Ciprofloxacin	5.9 (13)	5.9 (13)	100
Trimethoprim	11.3 (18)^b^	8.1 (18)	100^b^
Trimethoprim/Sulfamethoxazole	9.9 (22)	7.7(17)	97.8

^a^Phenotypic: phenotypic resistance of the 222 studied isolates; Genotypic: genotypic resistance of the isolates; Concordance: % of similarity between phenotypic and genotypic resistance

^b^Phenotypic screens of 62 isolates for trimethoprim resistance were not done, thus, reducing the total number of isolates tested for trimethoprim resistance to *N* = 160

## Discussion

Infections caused by NTS remain a significant contributor to childhood morbidity and mortality in SSA [1, 36]. Our study investigated the hypothesis of possible anthroponotic transmission of the evolved African *Salmonella enterica* ST313 by identifying NTS reservoirs in humans, animals, and the environment. This approach aimed to shed light on bacterial adaptation within specific host environments. Additionally, we searched for reservoirs of other NTS sequence types to gain a broader understanding of their distribution in these parts of Africa.

With a focus on one country from West Africa and one country from East Africa, we investigated reservoirs and potential transmission pathways of NTS in these parts of the world. Through extensive sampling, with almost 10,000 samples collected, and by using a One Health approach, we describe the frequencies and distribution of NTS, while also informing on AMR. Despite a few limitations, such as 1) low NTS isolation frequencies in certain sources under investigation (that might not allow for drawing concrete conclusions from this data), and 2) the fact that we only sampled children and specific geographic areas in each country (that might not be representative of the total population or the entire countries), we provide valuable insights into the molecular epidemiology of *Salmonella enterica* in diverse ecological niches. This study comprehensively contributes to a deeper understanding of NTS dynamics in SSA.

### Diverse serovar distribution across different sample sources with minimal serovar overlap

The study identified *Salmonella enterica* in each sample category investigated, all presenting possible transmission reservoirs for infections. We found a significantly diverse range of *Salmonella* serovars across the sample categories and in the two countries under investigation, with 59 different serovars amongst the 222 isolates found. The serovar distribution varied widely in samples other than blood cultures, with certain serovars predominantly found in specific sample types or geographic locations. Previous studies on the continent have also highlighted the broad serovar distribution in particular in livestock harbouring serovars not typically found amongst humans suggesting that agricultural animals may not be the primary contributors to NTS disease in humans [23, 27, 37, 38]. This broad distribution of serovars in livestock and in the environment highlights the complex ecology of *Salmonella enterica* transmission. Environmental samples, including dust and soil, showed considerable NTS contamination. This underscores the importance of environmental surveillance in understanding the ecology of *Salmonella enterica* transmission and the potential for soil and dust to serve as NTS reservoir for human and animal infection. This is particular true for serovars other than those causing invasive disease while still having the potential to cause diarrhoeal disease in humans.

As expected, the serovar *S*. Typhimurium was most predominant in blood samples from febrile children followed by *S.* Dublin and *S.* Enteritidis; in fact, the two latter serovars were only found in blood samples.

Other studies conducted on the continent over the past decade have also reported comparable results regarding the distribution of serovars contributing to iNTS disease in Africa. The serovar *S*. Dublin was more frequently isolated from patients with invasive disease in the study area of Ghana than elsewhere on the continent [30, 39, 40].

Interestingly, the overall carriage of NTS among children without diarrhoea was higher than in children with diarrhoea. In contrast to *S.* Typhi, the role of NTS stool carriage as a potential transmission reservoir is unclear and so far, NTS stool excretion has not been extensively studied in children with invasive disease [12, 33, 41]. However, asymptomatic carriage of *Salmonella enterica* and possible anthroponotic transmission, mainly within households, as a plausible mode of dissemination has been suggested for iNTS in Africa [37]. Serovar overlaps of stool and blood samples were also observed in the current study.

For example, *S*. Typhimurium ST 19 was not only found in blood cultures but also in children with and without diarrhoea, in livestock and, in the environment. We also identified overlap for *S*. Enteritidis, with most cases found in blood while one found in a patient with diarrhoea.

### Co-occurrence of specific virulence genes amongst all invasive serovars

Co-occurrence analyses revealed that all *S*. Typhimurium, *S*. Dublin and *S*. Enteritidis possessed the *spv* virulence gene cluster contributing to the invasive potential of the bacteria. All co-occurring genes were located on virulence plasmids specific for each serovar. This is in line with previous findings [35].

### Overall high genetic diversity of NTS and supporting evidence of a human reservoir of ST313

Within the *S*. Typhimurium serovar, distinct subclades were identified by detailed genomic and plasmid analyses, and included ST313 L2, similar to what was reported from Malawi [9] and ST19. Most common in causing invasive disease was ST19 followed by ST313, followed by the ST11 serovar *S*. Enteritidis and ST10 of the *S*. Dublin lineages. Similar patterns were already described in other countries of SSA. Not in line with our findings and with most reports from countries on the continent is the observation that in recent years ST313 seems to have been almost replaced by ST19 in this part of Ghana and Tanzania. ST19 has been described in much lower prevalence’s on the continent [42, 43] but there are more recent reports from Kenya with comparable findings to ours [9]. Interestingly, ST313 isolates displayed low genetic diversity and ST313 types were only found in human blood cultures and in human stool. This indicates a potentially significant role for stool carriage, where silent carriage could act as a source for infections. These findings further support the evidence of the solely anthroponotic transmission of the *Salmonella* serovar *S*. Typhimurium sequence type 313 as compared to ST19. The higher diversity observed within ST19 suggests a more heterogenous population with a broader host range as was seen in the present study. The population structure of *S*. Enteritidis exhibited a higher diversity than the genetically more homogenous *S*. Dublin clade, which might point to an outbreak scenario with *S*. Dublin cases.

### Antibiotic resistance high in isolates obtained from Ghanaian blood cultures and MDR linked to *S. Typhimurium* ST313

The study also investigated AMR amongst *Salmonella enterica* isolates, revealing varying levels of resistance across sample categories and countries. Resistance to commonly used antibiotics, such as ampicillin, chloramphenicol, and trimethoprim/sulfamethoxazole, was observed, albeit at different frequencies. MDR strains have consistently been a cause for concern, as evidenced by their prevalence in Ghanaian blood cultures during the earlier study period. These strains were predominantly associated with *S*. Typhimurium of the ST313 lineage 2. AMR surely contributed to the success of this particular lineage in SSA and MDR has previously been linked to this particular sequence type [44]. However, the currently prevailing ST19 lineage shows the absence of such MDR determinants, which is a positive development. Nonetheless, concerning is the emerging resistance to fluoroquinolones, an essential drug for treating various infections in SSA, associated with the serovar *S*. Enteritidis. Additionally, this study has identified colistin resistance linked to *S*. Dublin, adding concerns to the overall picture of AMR. Colistin is often considered a last-resort antibiotic for the treatment of infections with MDR bacteria. Thus, the emergence of resistance to it further narrows the already limited treatment options. Previous reports have shown a degree of intrinsic resistance to colistin for some *Salmonella enterica* serovars belonging to group D such as for example the serovar *S*. Dublin [45, 46]. And not overlooking a critical aspect, livestock and the environment might still present reservoirs for infections as was shown for example for *S*. Kentucky ST198 found in both niches. Thus, the interconnectedness between humans, animals and the environment should be recognized.

## Conclusion

In summary, our study reinforces the evidence of an exclusive human reservoir of ST313, and particularly concerning is its association with MDR. There remains a crucial need for further investigation into aspects such as stool carriage of *Salmonella* enterica, not only among patients with invasive disease, but also in asymptomatic individuals who might sustain invasive disease in communities. Additionally, our research offers comprehensive insights into the genetic diversity, distribution, and into possible transmission dynamics of NTS in Ghana and Tanzania. All sources under investigation presenting potential transmission reservoirs and harbouring AMR. These findings highlight the complex interplay between host specificity, genetic variability, and transmission patterns, emphasizing the necessity for integrated One Health approaches to effectively manage *Salmonella* infections. Moving forward, continued research into the factors driving the dissemination and evolution of *Salmonella enterica* is essential to inform targeted interventions and strategies for disease prevention and control.

## Supplementary Information

Supplementary Material 1.

## Data Availability

The dataset generated and/or analysed during the current study and results of genetic analyses are available as supplementary file (Excel file, Tab. 1–6). The raw sequence data reported in this paper have been deposited in the Genome Sequence Archive (Genomics, Proteomics & Bioinformatics 2021), Beijing Institute of Genomics, Chinese Academy of Sciences that are publicly accessible with the following accession number: CRA020362.
